# Changes in nucleosome formation at gene promoters in the archiascomycetous yeast *Saitoella complicata*

**DOI:** 10.3934/microbiol.2017.2.136

**Published:** 2017-03-16

**Authors:** Hikaru Nakamiya, Saeka Ijima, Hiromi Nishida

**Affiliations:** Department of Biotechnology, Toyama Prefectural University, Imizu, Toyama 939-0398, Japan

**Keywords:** archiascomycete *Saitoella complicata*, enlarged cell, histone acetylation, nucleosome position at gene promoter, trichostatin A

## Abstract

We measured the degree of nucleosome formation at the gene promoters in trichostatin A-treated (1, 2, and 3 µg/mL) cells of the archiascomycete *Saitoella complicata* and those in enlarged *S. complicata* cells after zymolyase treatment. TSA-treated and enlarged cells showed similar changes in nucleosome occupancy in five out of six positions in the gene promoters. These results suggest that changes in nucleosome formation at the gene promoters could serve as stress response mechanisms elicited in response to spheroplast (zymolyase treatment) and TSA treatment. In addition, we demonstrated that changes in nucleosome position occurred mainly in cells treated with 1 µg/mL TSA, whereas cells treated with 2 and 3 µg/mL TSA did not exhibit significant changes in the degree of nucleosome formation.

## 1. Introduction

Nucleosomes contain histone octamers around which DNA is wrapped [1]. Neighboring nucleosomes are separated by unwrapped linker DNA. Generally, a nucleosome's position with respect to the gene promoter plays an important role in yeast gene expression [2]–[5]. Nucleosome arrangement is also specific to an organism [6].

Trichostatin A (TSA) is a histone deacetylase inhibitor that promotes histone acetylation, which induces hyperacetylation of histones [7]. TSA influences nucleosome structure via histone acetylation. In addition, TSA influences nucleosome positions in the filamentous ascomycete *Aspergillus fumigatus* [8]. The acetylation and deacetylation of histones play an important role in the regulation of transcription [9]. Our previous study showed that TSA influences gene expression and nucleosome position in the archiascomycete *Saitoella complicata* [10]. Our study identified a total of 154 genes upregulated in a concentration-dependent manner in response to TSA treatment, whereas 131 genes were identified to be increasingly downregulated with increasing TSA concentration [10]. Most of nucleosome positions did not change after TSA treatment [10]. The anamorphic and saprobic budding yeast *S. complicata*, which is classified under Taphrinomycotina, represents the earliest ascomycetous lineage [11],[12]. The fission yeast *Schizosaccharomyces* is also classified under Taphrinomycotina [12].

In the previous study, we compared the nucleosome positions in 0 and 3 µg/mL TSA [10]. Thus, it was uncertain whether nucleosome position changed in a TSA concentration-dependent manner or not. If nucleosome position did not change in a TSA concentration-dependent manner, at which concentration did the position change? In this study, we investigated whether genes that are known to be regulated in response to TSA treatment also exhibit changes in nucleosome formation at the gene promoters in a TSA concentration-dependent manner.

In addition, the ascomycetous yeast *Saccharomyces cerevisiae* spheroplast was reported to enlarge using zymolyase [13],[14]. Enlarged spheroplast cells contain multiple nuclei [13]. It was uncertain how the multiple nuclei were maintained. Do nucleosome positions differ in between single nucleus and multiple nuclei? In bacterial enlarged spheroplasts, DNA was replicated and stress response genes were upregulated [15]. We found that *S. complicata* cells enlarge when grown in minimal SD broth (Takara, Japan) after zymolyase treatment. Thus, we measured the extent of nucleosome formation at the gene promoters in enlarged *S. complicata* cells and compared them with nucleosome formation levels in TSA-treated cells.

## 2. Materials and Method

### 2.1. *Saitoella complicata* culture

*Saitoella complicata* NBRC 10748 (= JCM 7358, = IAM 12963; type strain) was cultivated in YM broth (yeast extract, 3 g/L; malt extract, 3 g/L; peptone, 5 g/L; dextrose, 10 g/L) at 25 °C for 24 h as a control sample. Afterwards, TSA (1, 2, and 3 µg/mL) was added to the *S. complicata* culture; cells were subsequently incubated at 25 °C for 24 h. For the enlarged spheroplast generation, *S. complicata* was grown in minimal SD broth (Takara, Japan) at 25 °C for 30 h. Harvested cells were centrifuged for 5 min at 3000 rpm and suspended in buffer containing 0.8 M sorbitol and 25 mM phosphate at 25 °C for 20 min. Zymolyase 20T (Seikagaku corporation, Japan) was added to the cell suspension; the cells were incubated at 37 °C for 60 min. *S. complicata* cells were harvested, centrifuged for 5 min at 3000 rpm, and cultured in minimal SD broth (adjusted to pH 7.5) at 25 °C for 4–7 days.

### 2.2. Nucleosomal DNA fragment isolation

Equal volumes of *S. complicata* culture and 2% formaldehyde were mixed and incubated for 10 min. Next, 5 mL of 1.25 M glycine was added to the resulting solution. *S. complicata* cells were collected, washed with 50 mM Tris-EDTA buffer (pH 8), and then suspended in zymolyase buffer (1 M sorbitol, 10 mM DTT, and 50 mM Tris-HCl, pH 8.0). Zymolyase (Seikagaku corporation, Japan) (50 U) was added to the cell suspension, and the resulting solution was incubated at 37 °C for 1 h. Cells were collected by centrifugation and suspended in 2.5 mL of zymolyase buffer, after which 1 U of MNase (Takara, Japan) was added. The resulting digestion solution was incubated at 37 °C for 30 min, and the reaction was stopped by adding sodium dodecyl sulfate to a final concentration of 1% and EDTA to a final concentration of 10 mM. Proteinase K solution (5 µL) was added to the solution, and the mixture was incubated at 56 °C for 1 h. DNA was phenol/chloroform-extracted, ethanol-precipitated, and treated with RNase (Nippon Gene, Japan). Nucleosomal DNA fragments were isolated via electrophoresis on 2% agarose gel. The mononucleosomal DNA band was excised and purified using the QIAquick Gel Extraction Kit (Qiagen, Germany).

### 2.3. Quantitative PCR

**Table 1.** Primers used in this study.

Target position	Forward (5′ to 3′)	Reverse (5′ to 3′)	Product size (bp)
2351	ggcaggcagtccaatagagt	gagatcaagaggggttcacg	103
2810_1	gcagtttaacgacgagaaggtt	cgcctcggtaataggtattcat	110
2810_2	ggacaagctcctggtcttcc	cccttcaaagcacctcaatc	110
3456	gagaagctaaccgagcaacttt	tggccaattgaacaaacgat	109
5676_1	tcagcgattccccaagttat	gatgagggcgtcgagttc	110
5676_2	gttcacgaggacagatcagg	ggagttcgaaccatctttataacttg	109
5676_0 (control)	gagcgggatgtctttgtgat	ctaggcagtcactgggatcg	99

In this study, we selected six nucleosome positions in the gene promoters (300 nucleotides upstream of the translational start site) of the following four locus tags: G7K_2351-t1, G7K_2810-t1, G7K_3456-t1, and G7K_5676-t1. G7K_2351-t1 and G7K_2810-t1 encode homologs to 19S proteasome regulatory subunit Rpn3 and 20S proteasome-component α6 subunit Pre5, respectively, and are known to be increasingly downregulated upon treatment with increasing concentrations of TSA [10]. G7K_3456-t1 encodes a homolog to anaphase promoting complex subunit Apc11, whereas the G7K_5676-t1 gene is not homologous to any *Schizosaccharomyces pombe* protein. G7K_3456-t1 and G7K_5676-t1 are genes that are both upregulated in response to TSA treatment in a concentration-dependent manner [10]. Table 1 and Supplementary Figure 1 list the primers used in this study. We selected the position 5676_0 as an internal control, which showed the same nucleosome formation level between the cells treated with 0 µg/mL and 3 µg/mL TSA (Supplementary Figure 1) [10]. PCR was performed using the following cycling conditions: 1 cycle of 95 °C for 600 s and 45 cycles of denaturation (95 °C for 10 s), annealing (55 °C for 10 s), and extension (72 °C for 15 s). After the extension, a melting curve cycle was performed from 60 °C to 95 °C at 0.1 °C/s to confirm the absence of non-specific bands. The quantification cycle (Cq) values were obtained using LightCycler Nano Software (Roche, Basel). We calculated the nucleosome formation level using the following formula: 2^(Cq value at the position 5676_0 − Cq value at each position)^.

We performed analysis of variance (ANOVA) and a pairwise *t* test with Holm's adjustment using R statistical software (http://www.r-project.org/).

## 3. Results and Discussion

**Figure 1.**
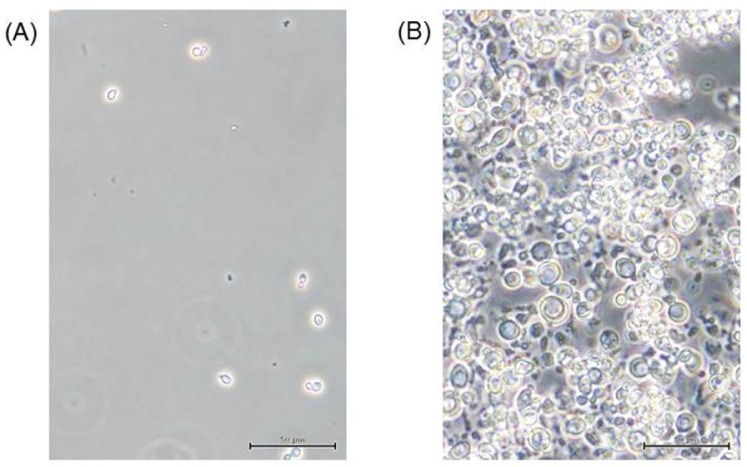
Phase contrast micrographs of *Saitoella complicata*. (A) Normal budding cells in minimal SD broth before zymolyase treatment. (B) Enlarged spheroplasts after 112 h of culture in minimal SD broth after zymolyase treatment. Phase contrast microscopy images were obtained using Olympus CKX41; bar = 50 µm.

The typical diameter of a *Saitoella complicata* cell is approximately 5 µm, whereas that of an enlarged spheroplast cultured in minimal SD broth after zymolyase treatment was measured to be approximately 15 µm (Figure 1).

**Figure 2.**
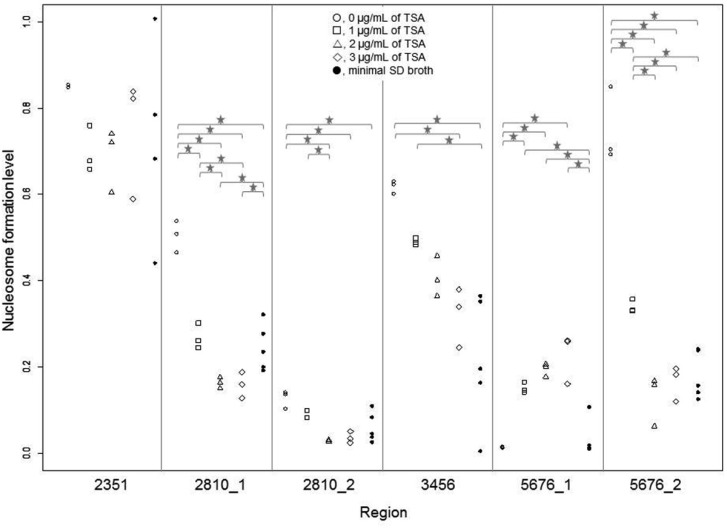
Comparison of nucleosome formation levels. The degree of nucleosome formation at position 5676_0 (control) is 1. We calculated the degree of nucleosome formation using the following formula: 2^(Cq value at the position 5676_0 − Cq value at each position)^. Star indicates *p* < 0.05 in a pairwise *t* test with Holm's adjustment.

ANOVA results showed that nucleosome formation levels were not significantly (*p* > 0.05) different at position 2351 but significantly different (*p* < 0.05) at the five other positions, namely, 2810_1, 2810_2, 3456, 5676_1, and 5676_2 (Figure 2).

Among the five positions, analysis using pairwise *t* test with Holm's adjustment showed no significant differences in terms of the degree of nucleosome formation at position 5676_1 (*p* > 0.05) between normal budding cells (0 µg/mL TSA) and enlarged cells (culture in minimal SD broth). However, significant differences (*p* < 0.05) in nucleosome formation levels were observed in the four other positions (2810_1, 2810_2, 3456, and 5676_2) (Figure 2). In addition, no significant differences in nucleosome formation were observed between enlarged cells and TSA-treated cells (2 and 3 µg/mL) at positions 2810_1, 2810_2, and 5676_2 (Figure 2). The above results strongly suggest that TSA-treatment and culture in minimal SD broth after zymolyase treatment exert similar effects on nucleosome formation at positions 2810_1, 2810_2, and 5676_2. Further research is necessary to confirm whether enlarged cells exhibit different histone acetylation patterns. Changes in nucleosome formation at the gene promoters can represent a stress response mechanism in cells subjected to spheroplast (zymolyase treatment) and TSA treatment. On the other hand, the degree of nucleosome formation at position 5676_1 was observed to be significantly different between enlarged cells and TSA-treated cells (Figure 2). However, nucleosome formation at this position was not significantly different between normal budding cells and enlarged cells. Thus, the observed nucleosome formation at position 5676_1 is specific to TSA-treated cells.

Changes in the degree of nucleosome formation appeared to occur in a TSA concentration-dependent manner at positions 3456 (decreasing) and 5676_1 (increasing) (Figure 2). However, no significant differences in nucleosome formation levels were observed between cells treated with 1 and 2 µg/mL TSA and between cells treated with 2 and 3 µg/mL TSA (Figure 2).

Nucleosome formation at position 5676_1 increased after TSA-treatment (Figure 2). On the other hand, nucleosome formation levels decreased after TSA-treatment at the neighboring position 5676_2 (Figure 2), which strongly suggests that a histone octamer can move from position 5676_2 to 5676_1. Based on the calculated nucleosome formation levels and *p* values, cells treated with 1 µg/mL TSA evidently showed nucleosome movement (Figure 2). Interestingly, changes in nucleosome position did not occur in enlarged cells, since nucleosome formation was observed only at position 5676_2 (Figure 2).

In positions 2810_1 and 2810_2 (neighboring regions), nucleosome formation levels decreased as a result of TSA-treatment (Figure 2). This suggests that two histone octamers may be absent at these two positions. The observed nucleosome depletion at position 2810_2 is inconsistent with the results of the previous study (Supplementary Figure 1) and suggests that the nucleosome occupancy at this position is unstable.

Except for position 2351, nucleosome formation levels in all other positions were significantly different between cells treated with 0 and 1 µg/mL TSA. However, no significant differences were observed between cells treated with 2 and 3 µg/mL TSA. The above results indicate that changes in the nucleosome formation occurred mainly in cells treated with 1 µg/mL TSA but not in cells treated with 2 and 3 µg/mL TSA.

## 4. Conclusion

We demonstrated that although TSA-treatment and zymolyase-treatment are completely different stimulus, TSA-treated cells and enlarged spheroplasts of *Saitoella complicata* showed similar changes in nucleosome formation in five out of six gene promoter positions examined in the present study. These results strongly suggest that changes in nucleosome formation could serve as a stress response mechanism of *S. complicata* cells. Different stressors (TSA and zymolyase treatments) induce similar changes in the patterns of nucleosome formation in gene promoters in *S. complicata*.
