# Supplementary material for: Changes in nucleosome formation at gene promoters in the archiascomycetous yeast Saitoella complicata
Source: AIMS Microbiol. 2017 Mar 16;3(2):136–42. doi: 10.3934/microbiol.2017.2.136 (PMC6605012; doi:10.3934/microbiol.2017.2.136)
Supplement: Supplementary file 1 [file microbiol-03-02-136-s001.pdf]

AIMS Microbiology

http://www.aimspress.com/journal/microbiology

AIMS Microbiology, 3(2): 136-142.
DOI: 10.3934/microbiol.2017.2.136
Received: 08 December 2016
Accepted: 09 March 2017
Published: 16 March 2017

Research article

Changes in nucleosome formation at gene promoters in the archiascomycetous yeast *Saitoella complicata*

Hikaru Nakamiya, Saeka Ijima and Hiromi Nishida *

Department of Biotechnology, Toyama Prefectural University, Imizu, Toyama 939-0398, Japan

* **Correspondence:** Email: hnishida@pu-toyama.ac.jp; Tel: +81-766-56-7500;
Fax: +81-766-56-2498.

Supporting Information

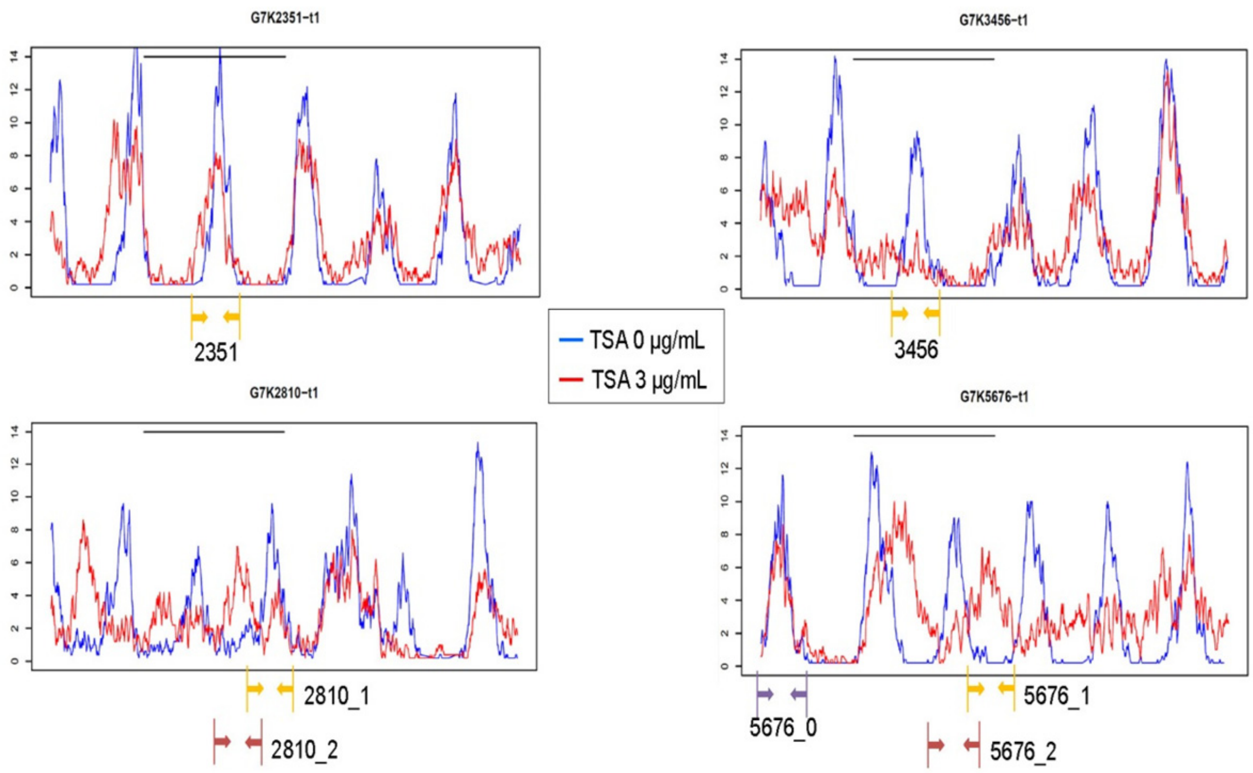

Supplementary Figure 1. Nucleosome position profiles within 500 nt upstream and downstream from the translation start site. Bar indicates the 300 nt upstream region from the translational start site. Blue and red indicate the nucleosome position profiles at 0 and 3 μg/mL TSA, respectively.

AIMS Press

© 2017 Hiromi Nishida, et al., licensee AIMS Press. This is an open access article distributed under the terms of the Creative Commons Attribution License (http://creativecommons.org/licenses/by/4.0)
